# Public Beliefs About Somatic Symptom Disorders

**DOI:** 10.3389/fpsyt.2018.00616

**Published:** 2018-11-20

**Authors:** Olaf von dem Knesebeck, Bernd Löwe, Marco Lehmann, Anna C. Makowski

**Affiliations:** ^1^Institute of Medical Sociology, University Medical Center Hamburg-Eppendorf, Hamburg, Germany; ^2^Institute and Outpatients Clinic for Psychosomatic Medicine and Psychotherapy, Hamburg, Germany

**Keywords:** somatic symptom disorder, mental health literacy, public beliefs, knowledge, survey, Germany

## Abstract

**Objectives:** Despite satisfactory effectiveness of treatment for somatic symptom disorders (SSD), treatment rates are low and treatment is usually initiated many years after first symptoms. In order to understand whether a lack of public mental health literacy might contribute to these poor treatment rates, we aimed to focus on two research questions: (1) What does the German public know and think about SSD? (2) Are knowledge and beliefs associated with socio-demographic factors and experiences with the disorder?

**Methods:** Two vignettes with symptoms of a SSD were used in a national telephone survey in Germany (*N* = 1,004). Vignettes differed regarding main type of symptom (pain vs. fatigue) and existence of an earlier somatic disease (yes vs. no). Respondents were asked concerning knowledge and beliefs about causes, symptoms, and treatment using a standardized questionnaire.

**Results:** 66% of the respondents agreed that a possible cause of the symptoms is a misinterpretation of body signals. About 1/3 recognized a mental health problem when confronted with the vignettes of cases with SSD. This rate was lower in case of a SSD with pain as the main symptom without a comorbid somatic disease (24.8%, 95%-CI: 21.1–28.6) compared to a case with fatigue as the main symptom and an earlier severe somatic disease (44.0%, 95%-CI: 39.6–48.3). Female respondents tended to have a more positive view on treatability and effectiveness of psychotherapy, while associations of knowledge and beliefs with education and age were inconsistent. Respondents who had contact with a person with SSD were more likely to think that psychotherapy is effective and that they know a lot about SSD compared to those who never had contact.

**Conclusions:** While most of the German public seems to be well informed about causes of SSD as well as treatability and the effectiveness of psychotherapy, the majority has problems with interpreting symptoms and does not think they know a lot about symptoms like that. Increasing knowledge about SSD by education interventions may help to promote adequate help seeking.

## Introduction

Public knowledge and beliefs about mental disorders can be conceptualized as “mental health literacy” (1). The concept is based on the more general term “health literacy” (2), initially defined as the ability to find, understand, and use medical information. Although health literacy has become an important concept in research and health policy, there is no consensus about the definition, and the conceptual model (3). Jorm et al. (4) introduced the concept of mental health literacy and defined it as “knowledge and beliefs about mental disorders which aid their recognition, management or prevention” (p.182). Accordingly, mental health literacy consists of several components, including the ability to recognize specific disorders, knowledge and beliefs about risk factors, causes, and professional help available as well as attitudes that facilitate appropriate help-seeking (5). A lack of public mental health literacy means that many members of the public do not know what they can do for prevention, people often delay or avoid seeking treatment, view recommended treatments with suspicion, or they are unsure how to assist people afflicted by mental health problems (6).

Although mental health literacy in general seems to be increasing (7), it has to be kept in mind that most respective studies and interventions focus on disorders like depression, schizophrenia, or anxiety (1, 8–10). These studies furthermore indicate that there are no consistent associations between mental health literacy and sociodemographic characteristics like gender and age (11, 12). In terms of education, some studies found inequalities in mental health literacy [e.g., (13)]. Also, previous contact to persons afflicted and own experiences with mental illness were associated with some aspects of mental health literacy (1, 14–17).

In contrast to the disorders mentioned above, there is not much known on the public knowledge and beliefs about somatic symptom disorders (SSD), formerly labeled as somatoform or functional disorders (18). The appropriate and timely provision of adequate treatment to patients suffering from SSD appears particularly problematic (19). Only 25% of affected patients undergo psychotherapy as the treatment with the best proven effectiveness (20), and the mean duration between onset of somatoform disorder and first psychotherapeutic or psychiatric treatment was estimated in primary care and secondary/tertiary care studies between 6 and 25 years (21, 22). Although physician-related barriers to the diagnosis and treatment of patients with somatoform disorders do exist (19, 23) and there is a risk of a somatization effect of a clinical consultation (24), the admission to hospitals of patients with functional and somatoform disorders strongly depends on health literacy and access to health care (25). Considering these data, it can be assumed that the general population may not be well informed about diagnosis and treatment of SSD, i.e., that SSD literacy may be low. However, to the best of our knowledge, public beliefs, and knowledge about SSD have not yet been investigated. In particular, it is not known whether the general public considers SSD as a mental disorder that is well treatable with psychotherapy (26), and as a disorder in which perceptual mechanisms such as somatosensory amplification are involved (27). Therefore, we focus on the following research questions: (1) What does the German public know and think about SSD? (2) Are knowledge and beliefs associated with socio-demographic factors and experiences with the disorder?

## Methods

### Study design and sample

Analyses are based on a national computer-assisted telephone survey conducted in November and December 2017. Sample consisted of adults aged 18 and older, living in private households in Germany. About 70% of the sample was randomly drawn from registered private telephone numbers. Additional computer-generated numbers allowed for inclusion of ex-directory households (landline numbers). The remaining 30% of the sample consisted of randomly generated mobile phone numbers (Random Digit Dialing). For a random selection of participants in the households, the Kish-Selection Grid was applied (28). Among mobile users, target person was the owner or main user of the mobile device. After having been informed that participation in the study is voluntary and that withdrawal from the study is possible at any time, 1,004 individuals participated, resulting in a response rate of 48.3%. Data collection procedure was approved by the Ethics Commission of the Medical Association in Hamburg (No. PV3707). Comparison with official statistics shows that age, gender, and education are similarly distributed in the sample and in the general adult population of Germany (Table 1).

**Table 1:** Distribution of sex, age, and education in the sample (*N* = 1,004) compared to official German statistics of the general population.

	**Sample**	**Official statistics**	p^*^
Sex (female, %)	51.9	50.7^1^	0.729
**LEVEL OF EDUCATION (%)**			
≤ 9 years	34.7	34.2^2^	0.441
10 years	31.1	32.0^2^
≥ 12 years	34.2	33.5^2^
**AGE (GROUPS, %)**			
18–24	9.4	9.2^3^	0.193
25–39	21.4	22.5^3^
40–59	33.8	35.6^3^
60–64	9.7	7.6^3^
> 65	25.7	25.1^3^

1 Federal Office of Statistics: Statistical Yearbook 2017, p 26 (Available online: https://www.destatis.de/DE/Publikationen/StatistischesJahrbuch/StatistischesJahrbuch.html. Last accessed April 23, 2018);

2 Federal Office of Statistics: Statistical Yearbook 2017, p, 84;

3 Federal Office of Statistics: Statistical Yearbook 2017, p, 31;

* *χ^2^-test: sample against official German statistics*.

### Vignettes

Before the interview, a vignette with signs and symptoms suggestive of a SSD was presented to the respondents. Two different vignettes were developed by the clinicians in our team (see Appendix). Both vignettes show cases of a SSD according to DSM-5 (18). The first sentence of the vignettes gives information about the A criterion (burdensome somatic symptom) and the C criterion (persistence of symptom burden) of SSD. The subsequent sentences consist of information regarding the B criterion concerning excessive thoughts about the severity of the symptoms, severe anxiety about ones health and the symptoms, and excessive symptom related behavior. Vignette A shows a case of a SSD with pain as the main symptom without a comorbid somatic disease, whereas vignette B shows a case with fatigue as the main symptom and an earlier severe somatic disease. Hence, the two vignettes differ in terms of main type of symptom and existence of an earlier somatic disease. Some studies indicate that socio-demographic characteristics may have an impact on public beliefs about mental disorders (11). Therefore, vignettes were additionally varied according to gender (female vs. male) and age (32 vs. 62 years), resulting in eight different vignettes that each were presented to about 125 respondents (i.e., about 12.5% of the sample). Both vignettes are also compatible with the bodily distress disorder according to ICD-11 (29) as they consist of cases with one or more distressing bodily symptoms and as they exemplify the excessive attention that the afflicted persons devote to the symptoms. However, we refer to SSD, because ICD-11 was not published at the time of data collection.

### Instruments

After the vignette was presented, respondents were asked five questions concerning their knowledge and beliefs about causes, symptoms, and treatment using a mainly standardized questionnaire. First, the respondents were asked about the possible diagnoses (“What's the matter with that person”?). Answers to this open-ended question were noted. Respondents who mentioned a mental health problem (e.g., psychosomatic disorder, burnout, depression, anxiety) were counted. Respondents' causal attribution for the symptoms presented was assessed by the following item: “A possible cause of these symptoms is a misinterpretation of body signals.” The item was coded from 1 (not true at all) to 4 (completely true). Furthermore, the respondents were asked whether the symptoms are treatable (1 = not at all to 4 = very well). This was followed by the question how effective psychotherapy is for the treatment of the symptoms. The scale again was ranging from 1 (not at all effective) to 4 (very effective). To summarize their knowledge, respondents were asked to agree or disagree with the following statement on a four-point scale: “I know a lot about symptoms like that.”

In terms of sociodemographic factors, age, gender, and education (highest educational degree) are introduced. To asses respondent's experience with symptoms described in the vignette, they were asked whether they themselves ever were afflicted by similar symptoms (yes vs. no). Moreover, they were asked whether they have or had personal contact to a person afflicted (yes vs. no).

### Analyses

The analyses were performed using SPSS 22. To analyze the knowledge and beliefs about the symptoms presented, the four-point scales were dichotomized by combining the first two and the last two response options (Table 2). Percentages are documented and compared between the two vignettes using Fisher's Exact test (research question 1). To analyze associations with knowledge and beliefs regarding the presented symptoms, sociodemographic variables and experiences with SSD are introduced into multiple logistic regression models (research question 2). The five dichotomized indicators of knowledge and beliefs are defined as dependent variables. Odds ratios, 95% confidence intervals, and Nagelkerke's R^2^ are displayed. *P*-values are reported for all analyses; values of *p* < 0.05 are regarded as statistically significant.

**Table 2:** Knowledge and beliefs about somatic symptom disorders.

	**Total sample (*N* = 1004)**	**Vignette A** **(pain without somatic disease, *n* = 499)**	**Vignette B (fatigue with previous somatic disease, *n* = 505)**	**P^1^**
Recognition of a mental health problem (% yes, 95%-CI)	34.5 (31.5–37.4)	24.8 (21.1–28.6)	44.0 (39.6–48.3)	<**0.001**
Misinterpretation of body signals as a cause (% rather agree/totally agree, 95%-CI)	66.4 (63.5–69.4)	67.3 (63.2–71.4)	65.5 (61.4–69.7)	0.584
Disorder is treatable (% well/very well, 95%-CI)	74.9 (72.2–77.6)	71.9 (68.0–75.9)	78.0 (74.4–81.6)	**0.024**
Psychotherapy is rather/very effective (%, 95%-CI)	80.5 (78.0–82.9)	82.0 (78.6–85.3)	79.0 (75.5–82.6)	0.255
I know a lot about symptoms like that (% rather agree/totally agree, 95%-CI)	30.7 (27.8–33.5)	27.2 (23.3–31.2)	34.1 (29.9–38.2)	**0.023**

1 *Fisher's exact test (X^2^) differences between vignettes A and B; CI, Confidence Interval. Significant differences (p < 0.05) are bold*.

## Results

Table 2 shows the distribution of knowledge and beliefs about SSD for the total sample and for the subsamples of the two vignettes. About 1/3 of the respondents recognized a mental health problem when confronted with the vignettes. This rate was lower in case of a SSD with pain as the main symptom without a comorbid somatic disease (24.8%), compared to a case with fatigue as the main symptom and an earlier severe somatic disease (44.0%). About 2/3 agreed that a possible cause of the symptoms is a misinterpretation of body signals, i.e., somatosensory amplification. Significantly fewer respondents thought that the case with pain and without a comorbid somatic disease is treatable, compared to the fatigue case with an earlier severe somatic disease. About 80% of the German public considered psychotherapy as a rather or very effective measure, while <1/3 thought, they know a lot about the symptoms presented.

Associations of beliefs with socio-demographic factors and experiences for vignette A (pain without somatic disease) are shown in Table 3. Age is negatively associated with the recognition of a mental health problem, i.e., the older the respondents the less often they recognized a mental health problem when confronted with vignette A. There are no significant associations of sociodemographic and experience-related factors with the belief that misinterpretation of body signals is a potential cause of this SSD. Women are more likely to think that this SSD is treatable than men. In terms of psychotherapy, older respondents and those with a low education are less likely, whereas respondents who had contact with a person with SSD are more likely to think that this measure is effective. Compared to the other beliefs, Nagelkerke's R^2^ is considerable higher in case of the subjective assessment of the own knowledge, indicating that sociodemographic and particularly experience-related factors in this case are more important for explanation.

**Table 3:** Associations with knowledge and beliefs about somatic symptom disorders (Vignette A, pain without somatic disease): Odds Ratios [95% Confidence Intervals]; significances.

	**Recognition of mental health problem**	**Misinterpretation of body signals as cause**	**Disorder is treatable**	**Psychotherapy is effective**	**I know a lot about symptoms like that**
Age in years	**0.987** **[0.975**–**0.999]** ^*^	0.991 [0.981–1.002]	1.001 [0.990–1.013]	**0.981** **[0.967**–**0.994]** ^**^	1.001 [0.988–1.015]
Female gender	1.330 [0.855–2.070]	0.928 [0.623–1.381]	**1.936** **[1.282**–**2.923]** ^**^	1.459 [0.897–2.373]	**2.028** **[1.213**–**3.390]** ^**^
Low vs. medium education	0.926 [0.520 1.651]	0.865 [0.517–1.445]	0.727 [0.428–1.233]	**0.492** **[0.262**–**0.923]** ^*^	1.040 [0.559–1.935]
Low vs. high education	1.333 [0.779–2.280]	0.818 [0.499–1.343]	0.909 [0.543–1.524]	0.639 [0.343–1.192]	0.606 [0.322–1.141]
Own affliction by SSD	1.529 [0.914–2.559]	1.218 [0.738–2.009]	1.210 [0.720–2.034]	0.607 [0.337–1.093]	**7.800** **[4.454**–**13.661]** ^***^
Contact to person with SSD	0.987 [0.631–1.544]	1.068 [0.710–1.606]	1.390 [0.911–2.122]	**2.185** **[1.298**–**3.678]** ^**^	**9.375** **[5.349**–**16.433]** ^***^
Nagelkerke R^2^	0.034	0.011	0.045	0.071	0.416

* p < 0.05,

** p < 0.01,

*** *p < 0.001. Significant associations (p < 0.05) are bold*.

Female gender, low education, and contact to a person afflicted are positively associated with the recognition of a mental health problem in case of fatigue with previous somatic disease (vignette B, Table 4). In terms of causal attribution, women are more likely to believe that misinterpretation of body signals is a potential cause of this SSD. Older respondents and respondents with a low education (compared to those with a medium education) are less likely to think that this SSD is treatable. The belief that psychotherapy is effective in the presented case of fatigue with previous somatic disease is negatively associated with age, while women, lower educated respondents and those who had or have contact to persons afflicted are more likely to hold this belief. Finally, the two experience-related factors are strongly linked to the subjective assessment of one's own knowledge.

**Table 4:** Associations with knowledge and beliefs about somatic symptom disorders (Vignette B, fatigue with previous somatic disease): Odds Ratios [95% Confidence Intervals]; significances.

	**Recognition of mental health problem**	**Misinterpretation of body signals as cause**	**Disorder is treatable**	**Psychotherapy is effective**	**I know a lot about symptoms like that**
Age in years	1.008 [0.997–1.020]	1.004 [0.992–1.015]	**0.986** **[0.974**–**0.999]** ^*^	**0.984** **[0.971**–**0.998]** ^*^	0.997 [0.984–1.010]
Female gender	**2.803** **[1.882**–**4.174]** ^***^	**1.577** **[1.063**–**2.339]** ^*^	1.092 [0.697–1.711]	**1.791** **[1.110**–**2.891]** ^*^	1.482 [0.954–2.303]
Low vs. medium education	1.036 [0.634–1.694]	0.657 [0.401–1.077]	0.621 [0.342–1.127]	0.899 [0.517–1.564]	1.259 [0.713– 2.223]
Low vs. high education	**3.182** **[1.920**–**5.273]** ^***^	1.125 [0.676–1.872]	**0.427** **[0.238**–**0.769]** ^**^	**2.197** **[1.161**–**4.160]** ^*^	1.538 [0.874–2.707]
Own affliction by SSD	1.192 [0.761–1.8761]	0.924 [0.589–1.449]	1.584 [0.899–2.790]	1.057 [0.612–1.826]	**5.167** **[3.175**–**8.408]** ^***^
Contact to person with SSD	**2.321** **[1.531**–**3.519]** ^***^	0.745 [0.490–1.134]	1.135 [0.711–1.814]	**1.905** **[1.176**–**3.088]** ^**^	**5.923** **[3.488**–**10.061]** ^***^
Nagelkerke R^2^	0.190	0.036	0.045	0.098	0.312

* p < 0.05,

** p < 0.01,

*** *p < 0.001. Significant associations (p < 0.05) are bold*.

## Discussion

Using two different SSD vignettes in a German general population survey, our results show that about 1/3 of the respondents recognized a mental health problem, about 2/3 agreed that a possible cause of the symptoms is a misinterpretation of body signals, and a large majority thought that the disorder is treatable (75%) and that psychotherapy is effective (80%). On the other hand, only 31% thought they know a lot about symptoms like that. Our results furthermore show, that female respondents tended to have a more positive view on treatability and effectiveness of psychotherapy, while associations of knowledge and beliefs with education and age were inconsistent. Respondents who had contact with a person with SSD were more likely to think that psychotherapy is effective and that they know a lot about SSD compared to those who never had contact. Additional analyses revealed that there are small and insignificant differences in public beliefs about SSD between the male and female vignettes as well as the young (32 years) and old (62 years) vignettes (results not shown in detail). There are two exceptions: More respondents agreed that misinterpretation of body signals is a potential cause of the symptoms when the person in vignette B was female (compared to the male vignette) and more respondents recognized a mental health problem when the person in vignette A was young (32 years old). Overall, however, there seems to be no consistent differences in beliefs about SSD depending on gender or age of the person afflicted.

In terms of the two vignettes, results indicate differences in public beliefs about SSD according to main type of symptom (pain vs. fatigue) and existence of an earlier somatic disease (yes vs. no). Significantly more respondents thought the disorder is treatable and they know a lot about such symptoms when confronted with the case of fatigue as the main symptom and an earlier severe somatic disease. Differences were most pronounced regarding interpretation of symptoms. Based on the vignettes used, it seems to be more difficult for the German public to recognize a mental health problem when pain compared to fatigue is the main symptom.

In a previous study (1), in which a vignette with signs of a depression was presented, about 72% of the German public recognized a depression, 80% thought this disorder is treatable, and 93% considered psychotherapy to be effective. Thus, recognition of disorder and beliefs about treatment differ between SSD and depression. These differences indicate that the observation of a generally improved mental health literacy (7) may not hold for SSD. As mentioned in the Introduction, most studies and interventions on mental health literacy focus on depression, schizophrenia, and anxiety (8–10), and this is the first study analyzing public knowledge and beliefs about SSD.

A review of Angermeyer and Dietrich (11) did not find consistent associations between mental health literacy and sociodemographic characteristics like gender and age. In our study, results concerning gender were more consistent across the two SSD vignettes, showing that female respondents tended to have a more positive view on treatability and effectiveness of psychotherapy. In terms of education, like in our analyses, some studies found that highly educated people more often recognize the mental health problem in question (1, 13). However, these inequalities were not consistent for all indicators of mental health literacy in our analyses as well as in the mentioned previous studies.

Moreover, previous contact to persons afflicted and own experiences with mental illness have been found to be associated with aspects of mental health literacy in some studies (1, 14–17). With regard to the results presented here, it is remarkable that the only significant difference between respondents who were afflicted by SSD and those who never had SSD was that the former stated to know more about the presented symptoms. Hence, affliction does not seem to matter for beliefs about treatment, recognition of a mental health problem, or identifying a correct cause of the symptoms. Against this background, our results do not support the assumption that people with a history of the disease in question or treatment experiences have beliefs closer to those of professionals (16).

Some methodological limitations have to be considered when evaluating our findings. Although various items measuring knowledge and beliefs about causes, symptoms, and treatment were used in the survey, we were not able to cover all aspects of SSD literacy. Moreover, all indicators relied on single-item measures. While these items mostly were used in previous studies (1, 13), they cannot be considered as sufficiently validated. Especially in terms of beliefs about causes and treatment effectiveness, it is difficult to doubtlessly evaluate which answer is “correct.” Furthermore, even though the use of vignettes as a stimulus is widespread and considered a strength in public mental health research, they have to be short to be included into surveys. As SSD is a complex disorder with three diagnostic criteria (30), it is unclear whether this disorder is adequately represented in short vignettes like ours. Furthermore, SSD is polymorphic and our analyses are restricted to two vignettes. Also, analyses on associations to some extent are crude because we decided to dichotomize the variables for the logistic regression models for the sake of clearness and comprehensibility. Finally, results are based on a German sample and cannot be generalized to other countries.

## Conclusions

While most of the German public seems to be well informed about causes of SSD as well as treatability and the effectiveness of psychotherapy, the majority has problems with interpreting symptoms and does not think they know a lot about symptoms like that. This finding can be interpreted as a lack of SSD literacy, at least in some important dimensions, and might contribute to the fact that only a minority of patients with SSD receive early and appropriate treatment. Increasing knowledge about SSD by education interventions may help to promote adequate help seeking.

## Author contributions

OvdK developed the research question and wrote the first draft of the manuscript. AM undertook the statistical analyses. BL, ML, and AM critically revised the manuscript and added important aspects. All authors were involved in the study design and in the development of the questionnaire. They also approved the final manuscript.

### Conflict of interest statement

The authors declare that the research was conducted in the absence of any commercial or financial relationships that could be construed as a potential conflict of interest.
