# Supplementary material for: Public Beliefs About Somatic Symptom Disorders
Source: Front Psychiatry. 2018 Nov 20;9:616. doi: 10.3389/fpsyt.2018.00616 (PMC6255972; doi:10.3389/fpsyt.2018.00616)
Supplement: Supplementary file 1 [file Data_Sheet_1.docx]

**Appendix**

Vignette A (pain without somatic disease)

Since one year, 32 years old/62 years* old Anja/Anton* S. has been suffering from constant piercing pain in the left side of her body, in her hand, arm, leg, and in her feet. She feels very restricted in her daily life. Medical examinations have provided no explanation for her complaints. Mrs. S has an intense fear for her health and is thinking about her complaints all the time. She is constantly surfing the internet to learn more about her troubles.

Vignette B (fatigue with cured somatic disease)

For more than six months, 32 years old/62 years old * Monika/Martin* E. has been suffering from total exhaustion, accompanied by chest pain, pain in her muscles and her shoulder. After a fully cured inflammation of the heart muscle eight months ago, she is continuously thinking that her complaints indicate a severe heart disease. According to a current medical examination, her heart is in a good condition. She still has intense fear regarding her health and her complaints. Mrs. E. is investing a lot of time and energy on her health concerns.

*Gender and age of the person in the vignettes were systematically varied.
